# Rethinking medical appointments

**DOI:** 10.51866/lte.501

**Published:** 2023-11-12

**Authors:** Khaled Moustafa

**Affiliations:** 1 PhD, The Arabic Preprint Server (ArabiXiv), Paris, France. Email: khaled.moustafa@arabixiv.org

**Keywords:** Appointment scheduling, Physician consultation, Patient satisfaction, Healthcare system, Unscheduled consultations

## Dear editor,

In many countries, medical appointments are the most common way to consult a physician outside of emergency services. Medical appointments offer various advantages for patients and physicians, including improved organisation, enhanced efficiency, better preparedness and continuity of care.^1^ However, despite these benefits, there are also several drawbacks associated with medical appointments that need to be highlighted and overcome.

One important issue is the prolonged waiting time, which can lead to frustration, stress and a sense of wasted time for patients. Appointments are typically scheduled days, weeks or even months ahead in certain cases, yet the consultation time itself may be limited to only a few minutes. This brevity can leave patients feel rushed and make it challenging for them to adequately express all their concerns. Waiting for weeks or months for an appointment that lasts only a few minutes may substantially impact the satisfaction and perceived value of the appointment among patients. This scenario may increase the risk of important details being overlooked, potentially leading to misdiagnoses, inadequate treatment plans and negative patient outcomes. When physicians prioritise numbers over patient well-being by allocating limited or fixed time for each consultation to accommodate a large number of patients, the healthcare system may fail to fulfil its mission appropriately.

Patient satisfaction is typically directly proportional to the consultation time but inversely proportional to the waiting time. A substantial increase in patient satisfaction scores is notably linked with a consultation time surpassing the initially anticipated timeframe.^2,3^ Patients who experience a short waiting time before consultation and a long consultation time tend to be more satisfied than do those who experience a long waiting time before consultation and a short consultation time.^4^

Delayed medical appointments may also prompt patients to seek urgent care services in hospitals even for minor symptoms, which can overwhelm these facilities, resulting in a decreased sense of urgency for patients who genuinely require immediate attention. Accordingly, more serious situations may lose priority.

Appointment scheduling can cause anxiety for patients, especially in urgent or time-sensitive situations. A prolonged waiting time can exacerbate stress levels in patients when immediate attention is required. Owing to time pressure, physicians may struggle to provide personalised attention and thorough examination that patients require, leading to feelings of anxiety and stress about the accuracy of their diagnosis or the effectiveness of the recommended treatments.

Finally, patients themselves may fail to attend their appointments, resulting in wasted time for both physicians and other patients.

It is worth considering a combination of scheduled and unscheduled medical appointments to address the abovementioned challenges. One approach could involve allocating half of the working week (e.g., 2–3 days) for scheduled appointments and the other half (e.g., 2–3 days) for free unscheduled consultations on a first-come, first-served basis. Alternatively, physicians could allocate their mornings for unscheduled consultations and their afternoons for scheduled appointments or vice versa. By adopting such an approach, physicians could accommodate both preferences and effectively address a range of situations that better suit their own and patients’ needs. Seeking consultations with physicians whenever needed is a more flexible approach than scheduling fixed appointments, as health issues requiring immediate medical attention may arise at any moment.

The waiting time may be shorter for unscheduled appointments than for scheduled appointments. Offering unscheduled consultations may then help patients save time.
